# Reconfigurable Coplanar Waveguide (CPW) and Half-Mode Substrate Integrated Waveguide (HMSIW) Band-Stop Filters Using a Varactor-Loaded Metamaterial-Inspired Open Resonator

**DOI:** 10.3390/ma11010039

**Published:** 2017-12-28

**Authors:** Juan Hinojosa, Adrián Saura-Ródenas, Alejandro Alvarez-Melcon, Félix L. Martínez-Viviente

**Affiliations:** 1 Department of Electronics and Computer Engineering, Universidad Politécnica de Cartagena, Plaza del Hospital No. 1, 30202 Cartagena, Spain; adriansaura92@gmail.com (A.S.-R.); felix.martinez@upct.es (F.L.M.-V.); 2 Department of Information and Communications Technology, Universidad Politécnica de Cartagena, Plaza del Hospital No. 1, 30202 Cartagena, Spain; alejandro.alvarez@upct.es

**Keywords:** coplanar waveguide (CPW), metamaterials, microwave filters, substrate integrated waveguide (SIW), tunable devices, varactor diode

## Abstract

An open ring resonator (ORR) loaded with a varactor diode is designed and implemented in order to achieve high-performance tunable band-stop filters in planar technology with a compact size. This varactor-loaded ORR (VLORR) is versatile. It allows a shunt connection with different planar waveguide sections. In this paper, it has been connected to a coplanar waveguide (CPW) and a half-mode substrate integrated waveguide (HMSIW). As a reverse bias voltage is applied to the VLORR, a continuous tuning over the resulting stop-band can be achieved. To illustrate the possibilities of the VLORR, three prototypes have been designed, fabricated, and characterized. The three prototypes show an outstanding performance, with a rejection level at the resonant frequency and a tuning range greater than 12 dB and 85%, respectively. This VLORR has high potential value in microwave communication systems to eliminate unwanted signals.

## 1. Introduction

In many emerging multiband communication systems, microwave frequency-tunable filters are required with advanced features in terms of functionality and reduced volume and complexity [1,2]. Thus, several strategies using, for example, micro-electromechanical systems, ferroelectrics, liquid crystal, and magnetic materials, or varactor diode-based technologies have been used to reach these requirements [3,4,5,6,7,8,9]. MEMS switches present high quality factor. However, their switching time is slower than the varactor diodes, and variations in capacitance are difficult to achieve. Ferroelectrics, magnetics, and liquids are interesting materials for the novel design of tunable microwave devices. Nevertheless, the manufacturing cost of these components is high compared to varactor diodes, which are low cost, tunable, and can be easily integrated with other devices in planar technology. Therefore, varactor diodes can be a good solution for the design of planar tunable filters when fast tuning speed and low fabrication cost are required. The large circuit sizes of the traditional planar filters [10], using shunt stubs or stepped-impedance lines, can be reduced by means of metamaterial resonators such as open versions of the split ring resonators (OSRRs) and complementary split ring resonators (OCSRRs) [11,12,13,14,15,16,17]. 

In communication systems, band-stop filters play an important role in helping to eliminate unwanted signals. An interesting example on the use of split ring resonators (SRRs) to obtain compact band-stop subsystems is [18], in which multiple notch bands are etched on printed circular patch antenna. Band-stop filters integrating complementary split ring resonators (CSRRs) in the ground plane of microstrip structures have also been proposed [19,20,21]. A recent publication describes an example of tunability in microstrip technology [22]. However, in contrast to microstrip, there has been not so much published research on tunable coplanar waveguide (CPW) [9,23,24,25,26,27] and tunable substrate integrated waveguide (SIW) [28,29,30] band-stop filters using varactor diodes. 

One of the interesting characteristics of the CPW with respect to microstrip components is that the surface-mounted elements as varactor diodes can be easily integrated thanks to their coplanar ground planes. The integration of SRRs with CPW structures has been proposed in previous works to conceive compact band-stop filters [31]. The tunable CPW band-stop filters proposed in [9,23,24,25] are inspired by shapes found on metamaterial structures, while the reconfigurable characteristics are achieved by means of varactor diodes as variable loads. Such filters exhibit good performance and behave as resonators connected or coupled to a CPW, or to a defected ground structure (DGS). However, these filters still require a large size and, when tuning the operating frequency, it is not possible to obtain a constant absolute bandwidth simultaneously with a wide tuning range. An interesting compact topology with a constant absolute bandwidth and a wide tuning range consists of modifying the electrical length of a bottom-side meander line by tuning loaded varactor diodes [26]. However, all these different configurations use more than one varactor diode. A possible solution to these drawbacks including a significant reduction in size is to use a modification of the SRR, called open split ring resonator (OSRR) [12,13], and load it with a varactor diode (VLOSRR) as in [27].

SIWs and half-mode substrate integrated waveguides (HMSIWs) are planar guided-wave structures with the same advantages than microstrip and CPW: low fabrication cost and easy integration with planar components. SIWs and HMSIWs offer insertion loss, quality factor, and power handling capability in microwave and millimeter wave applications better than conventional microstrip or coplanar waveguides [32,33,34,35,36]. The tunable SIW band-stop filters presented in [28,29,30] use varactor diodes as tuning elements and are based on ridged and half-wavelength resonators, with embedded split ring resonators. These filters have a large size. Their tuning ranges are lower than 16%, with a constant absolute bandwidth, except for the solution presented in [30], which reaches up to 70% tuning range, but with a non-constant absolute bandwidth, and they use several cascaded varactor-loaded cells. Other topologies [37,38] using varactor diodes are based on substrate integrated coaxial-cavity resonators. These structures implement tunable two-pole band-stop filters and use microstrip lines or CPWs to excite the coaxial-cavity resonators. They need many varactor diodes and their sizes are large. Their tuning ranges are higher than 40%, but with a non-constant absolute bandwidth. The use of SRRs integrated in SIW structures to design compact filters without reconfigurable characteristics has been introduced in the past [39,40,41,42,43,44,45]. These filters are mainly loaded with SRRs or CSRRs [11,16], due to the difficulty of introducing a version of the open split ring resonator (OSRR) [12] within a SIW structure. This is not the case for the HMSIW, since an interesting solution has been recently proposed for the compact design of band-stop filters using an open ring resonator (ORR) [46].

In this paper, novel compact tunable CPW and HMSIW band-stop filters, allowing a wide tuning of the resonant frequency simultaneously with a constant absolute bandwidth, are presented. Both CPW and HMSIW structures use a novel cell, called varactor-loaded open ring resonator (VLORR). The VLORR is composed of an open ring resonator (ORR) and a varactor diode as a tuning element. The ORR consists of an open ring aligned with an open window etched in the ground plane of the planar waveguide. The varactor diode is simply connected between the open ring and the ground. The VLORR cell is versatile, since it can be easily connected to a parallel section of a planar waveguide such as CPW and HMSIW. At the resonant frequency of the ORR, a transmission cero occurs. The varactor diode loaded to the ORR provides a suitable variable capacitance as a reverse bias is applied to it and, therefore, allows for variation of the resonant frequency to the desired position within the operational frequency band. In comparison with the VLOSRR cell used in the tunable CPW band-stop filter presented in [27], the proposed VLORR cell has a different structure and working principle. Both cells use a varactor diode. However, the ORR has one ring instead of two for the OSRR. Consequently, the equivalent circuit of the ORR has a connection to ground through a radiation resistance at the resonant frequency, while the OSRR is directly connected to ground through the coupling to the second ring. Unlike the OSRR (VLOSRR), which performs as a pass-band or stop-band depending on whether it is connected to a microstrip or CPW section [12,13,27], the proposed ORR (VLORR) has the same stop-band behavior independently on how it is inserted within a microstrip, CPW, or HMSIW section.

This paper is organized as follows. The structure, equivalent circuit and analysis of the CPW and HMSIW band-stop filters loaded with a VLORR cell are described in Section 2. This section also includes a study of the undesired radiation effects. Experimental results obtained by means of first- and second-order tunable VLORR-CPW band-stop filters and a first-order tunable VLORR-HMSIW band-stop filter are discussed and compared with other tunable CPW and SIW band-stop filters in Section 3. Finally, conclusions are presented in Section 4.

## 2. Tunable CPW and HMSIW VLORR-Band-Stop Filters: Analysis and Design

### 2.1. Tunable CPW and HMSIW VLORR-Band-Stop Filters: Structures and Equivalent Circuit Models

**Figure 1:**
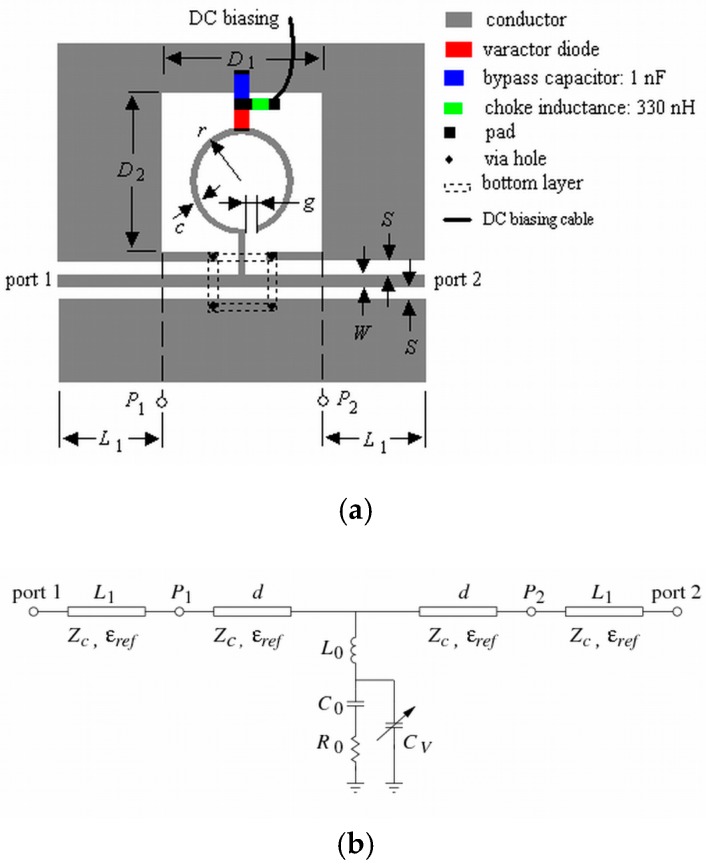
CPW loaded with a VLORR (**a**) structure and (**b**) simplified equivalent circuit.

**Figure 2:**
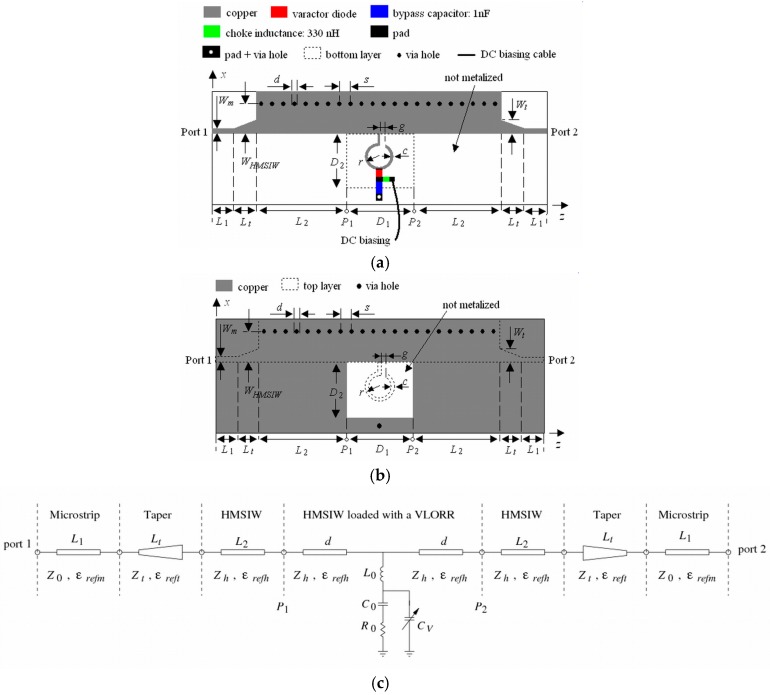
HMSIW loaded with a VLORR (**a**) top structure, (**b**) bottom structure and (**c**) simplified equivalent circuit.

The structures and equivalent circuit models of the proposed tunable CPW and HMSIW band-stop filters are shown in Figure 1 and Figure 2, respectively. Both structures use a basic open ring resonator (ORR) [22,46] loaded with a varactor diode. This cell, called varactor-loaded open ring resonator (VLORR), is connected to a section of a CPW or HMSIW line. In Figure 1a, the ORR is printed in an open window $D_{1}\times D_{2}$, located in one of the two lateral ground planes of the CPW line. The two lateral ground planes are interconnected by means of four via holes and four metallic strips printed in the bottom substrate layer, to avoid the excitation of parasitic modes. The VLORR cell is connected to the input port 1 and output port 2 by means of two sections $L_{1}$ of CPW line. Unlike the CPW structure, the HMSIW structure has the open window $D_{1}\times D_{2}$ in the bottom side of the HMSIW section (Figure 2b). To carry out measurements with the HMSIW structure (Figure 2a), a microstrip line $L_{1}$ and a tapered microstrip section $L_{t}$ have been added in both ports. The tapered microstrip section $L_{t}$ with two different widths ($W_{m}$, $W_{t}$) realizes the transition between the microstrip line $L_{1}$ of width $W_{m}$ and the HMSIW line $L_{2}$ of width $W_{HMSIW}$. This transition is used to transform the quasi-TEM mode of the microstrip line into the ${\mathrm{TE}}_{10}$ mode in the HMSIW line. Therefore, the VLORR cell is connected to the input port 1 and output port 2 through two HMSIW lines, two tapered microstrip sections, and two microstrip lines of lengths $L_{2}$, $L_{t}$, and $L_{1}$, respectively. Both structures have the cathode of the varactor diode placed at the middle point of the open ring and the anode connected to ground by means of a bypass capacitor. The capacitance of the varactor diode is controlled by means of a reverse DC bias voltage. This is applied through a DC bias network, performed with a choke inductance and a bypass capacitor.

The simplified equivalent circuits of the CPW and HMSIW structures loaded with a VLORR cell are depicted in Figure 1b and Figure 2c, respectively. They were obtained from previous electromagnetic (EM) analysis of their frequency responses, taking into account the initial simplified model of the ORR cell [22,46], with a variable capacitance $C_{V}$, as the ideal model of a varactor diode. The ORR cell is modeled by means of a shunt series $R_{0}$ $L_{0}$ $C_{0}$ resonant circuit connected between two CPW sections or two HMSIW sections of length $d=D_{1}/2$. The VLORR cell consists of the equivalent circuit of the ORR cell with a variable capacitance $C_{V}$ in parallel with the series $R_{0}$ $C_{0}$ circuit. From the equivalent circuit of the VLORR and omitting $R_{0}$, we can identify a resonant frequency $f_{0}=1/(2\pi \sqrt{L_{0}\;C_{T}})$ leading to a transmission zero. This resonant frequency $f_{0}$ can be varied in a controlled way through the total capacitance $C_{T}=C_{0}+C_{V}$ of the VLORR cell, which is the contribution of the fixed capacitance $C_{0}$ of the ORR cell and the variable capacitance $C_{V}$ of the varactor diode. Therefore, this diode works as a tuning component and its applied reverse DC bias voltage acts as control signal.

### 2.2. CPW and HMSIW Band-Stop Filters Using ORR

The initial analysis and design of the proposed tunable CPW and HMSIW band-stop filters can be started by omitting the varactor diode and the DC polarization network in Figure 1a and Figure 2a. Then, the equivalent circuits of these band-stop filters loaded with a basic ORR cell are the same as Figure 1b and Figure 2c without the variable capacitance $C_{V}$.

The basic ORR cell is a modified open version of the open interconnected split ring resonator (OISRR) [14]. The series $R_{0}$ $L_{0}$ $C_{0}$ resonant circuit (Figure 1b and Figure 2c) models the open ring. In the case of the CPW structure, the open ring is printed in an open window $D_{1}\times D_{2}$ located in one of the two lateral ground planes of the CPW and is connected at a point of the central conductor (Figure 1a), while the HMSIW structure has the open ring connected to a point of a HMSIW section, and the open window $D_{1}\times D_{2}$ is just etched in the bottom wall (Figure 2b). As mentioned before, between the shunt series $L_{0}$ $C_{0}$ resonant circuit and ground there is a series resistance $R_{0}$, which models the two possible contributions to the losses of the structure: radiation and ohmic. The ohmic losses in the metal ($R_{c}$) depend on the dimensions (*r*, *c*, and *g*) of the open ring, while the radiation losses ($R_{r}$) are determined by the size $D_{1}\times D_{2}$ of the open window.

**Figure 3:**
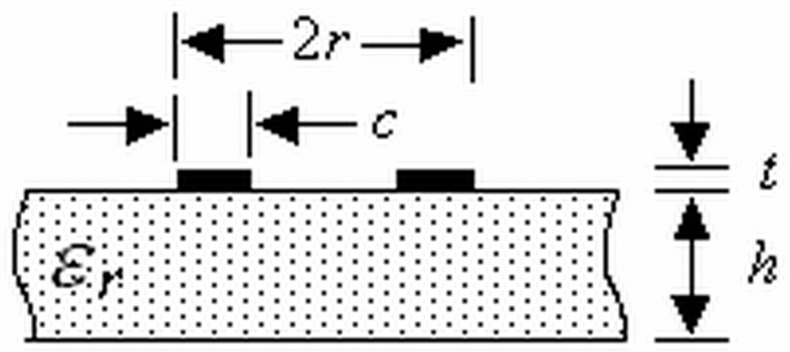
Cross section of a basic ORR cell.

$R_{0}$, $L_{0}$, and $C_{0}$ can be determined from previous electromagnetic (EM) simulations as in [46] or by using the derived equations shown below. Approximate equations for $L_{0}$ and $C_{0}$ were obtained from [47] and a Schwartz-Christoffel transformation of the cross section of the basic ORR cell (Figure 3). The expressions that result from this analysis depend on the design parameters of the ring. On the other hand, $R_{0}$ is calculated from the unloaded quality factor $Q_{u}$ and return loss at $f_{0}$:

(1) $$L_{0}=\mu_{0}r(\log(8r/(t+c))-0.5)/\alpha$$

(2) $$C_{0}=\alpha (2\pi r)\varepsilon_{0}\varepsilon_{ef,ORR}K(k^{\prime })/K(k)$$

(3) $$Q_{u}=\omega_{0}L_{0}/R_{0}=f_{0}/\Delta f_{0}/(1-{\left|S_{11}\right|}_{f0})$$

where $r_{0}=r-c/2$, $\varepsilon_{ef,ORR}=1+\frac{\left(\varepsilon_{r}-1\right)}{2}\frac{K(k^{\prime })}{K(k)}\frac{K(k_{1})}{K(k_{1}^{\prime })}$, k=(r−c)/r, $k_{1}=\frac{\sin h(\pi (r-c)/2/h)}{\sin h(\pi r/2/h)}$, $k^{\prime }=\sqrt{1-k^{2}}$, $k_{1}^{\prime }=\sqrt{1-k_{1}^{2}}$, α=−0.768c+1.5462 (*c* in mm), $K(k^{\prime })/K(k)$, and $K(k_{1})/K(k_{1}^{\prime })$ ratios can be calculated approximately from analytical relationships defined in [48]. The relative errors with respect to a commercial electromagnetic (EM) simulations (Ansys HFSS) for the resonant frequency ($f_{0}$) and 3 dB stop-band bandwidth ($\Delta f_{0}$) are lower than 3% for 1.8 ≤ *r* (mm) ≤ 2.6, 0.1 ≤ *c* (mm) ≤ 0.3, $\varepsilon_{r}$ = 10.2, *g* = 0.2, and $D_{1}\times D_{2}=4r\times 4r$.

**Figure 4:**
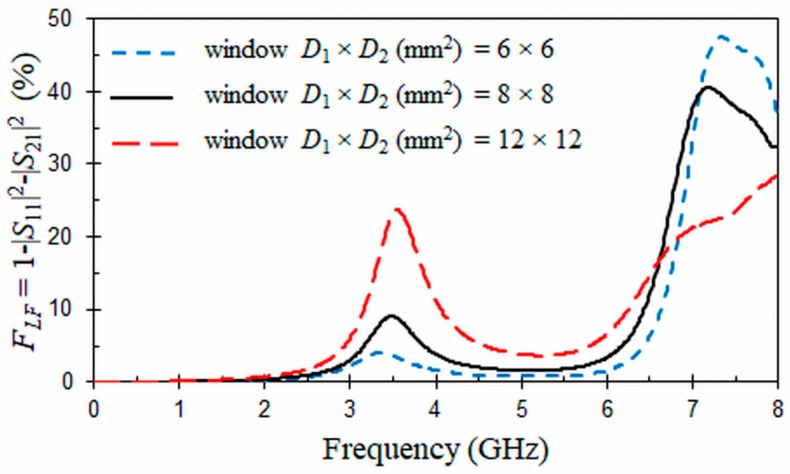
Forward loss factors (in %) for the proposed CPW band-stop filter as a function of frequency and different open windows $D_{1}\times D_{2}$. Dimensions (mm) and permittivity: *r* =1.9, *c* = 0.3, *g* = 0.2, *W* = 0.374, *S* = 0.163, *L*_1_ = 5.5, *h* = 0.635, and *ε_r_* = 10.2 (tg*δ* = 0).

**Figure 5:**
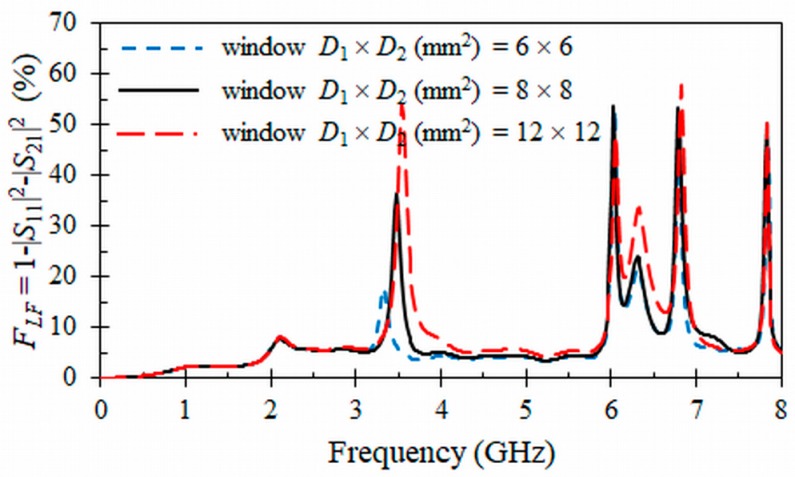
Forward loss factors (in %) for the proposed HMISW band-stop filter as a function of frequency and different open windows $D_{1}\times D_{2}$. Dimensions (mm) and permittivity: *r* = 1.9, *c* = 0.3, *g* = 0.2, *W_HMSIW_* = 11.65, *s* = 1.2, *d* = 0.6, *W_m_* = 0.594, *W_t_* = 4.66, *L*_1_ = 5, *L*_2_ = 11, *L_t_* = 28.95, *h* = 0.635, and *ε_r_* = 10.2 (tg*δ* = 0).

The ORR inserted in the window $D_{1}\times D_{2}$ opened in the CPW and HMSIW structures can cause radiation loss. Thus, we have studied the radiation effects of these structures (Figure 4 and Figure 5) as a function of frequency and different sizes of the open window $D_{1}\times D_{2}$ through EM simulations and the forward loss factor:

(4) $$F_{LF}=1-{\left|S_{11}\right|}^{2}-{\left|S_{21}\right|}^{2}$$

In the loss factors presented in Figure 4 and Figure 5, only losses due to undesired radiation effects are considered, since metallic and dielectric losses were neglected in the corresponding EM simulations, which were obtained by means of a commercial simulator (HFSS, ANSYS, Canonsburg, PA, USA). Both CPW and HMSIW structures have been simulated on a substrate with a relative permittivity $\varepsilon_{r}=10.2$ (tg*δ* = 0) and thickness *h* = 0.635 mm. The dimensions of the CPW and HMSIW structures loaded with an ORR are included in the captions of Figure 4 and Figure 5. They were optimized to have a resonant frequency at $f_{0}$ = 3.5 GHz with a 3 dB stop-band bandwidth of $\Delta f_{0}$ = 0.4 GHz and a characteristic impedance around $Z_{0}$ = 50 Ω by using Equations (1)–(3) and analytical relationships [49,50]. The dimensions of the HMSIW was adjusted to have a cut-off frequency at $f_{c}$ = 2 GHz [36]. In Figure 4, it can be seen that radiation losses for the CPW structure loaded with an ORR present two peaks. One of the two peaks appears at the resonant frequency $f_{0}$ = 3.5 GHz of the ORR and has a direct relation with the size of the open window, decreasing as this window becomes smaller. The second peak is at frequency $f_{s}$ = 7 GHz, which is two times the resonant frequency $f_{0}$ and corresponds to a spurious resonance. The relation of the intensity of this spurious resonance with the size of the open window is opposite to the main resonance, increasing as the size of the window decreases. Therefore, when the window has the minimum size of $D_{1}\times D_{2}=6\times {6\;\mathrm{mm}}^{2}$, the radiation losses at the frequency of resonance $f_{0}$ have a minimum value of 4.1%, while radiation at the spurious resonance is at its maximum, with a value of 47.5%.

On the other hand, radiation losses for the HMSIW structure loaded with an ORR (Figure 5) present up to six peaks in the same frequency range as CPW structure. The first peak appears at the cut-off frequency $f_{c}$ = 2 GHz of the HMSIW. The second is located at the resonant frequency $f_{0}$ = 3.5 GHz of the ORR. The third and the following peaks are due to higher-order modes in the HMSIW structure and are considered as spurious band. The different sizes of the open window have little effect on radiation losses of the ORR cell for the first peak, which are lower than 8%. At the resonant frequency $f_{0}$, the same trend as for the CPW structure is observed. The radiation losses at the resonant frequency $f_{0}$ have a minimum value of 17.7% when the window has the minimum size ($D_{1}\times D_{2}=6\times {6\;\mathrm{mm}}^{2}$). The HMSIW structure exhibits higher radiation losses than the CPW structure, probably due to long microstrip-to-SIW transitions. 

**Figure 6:**
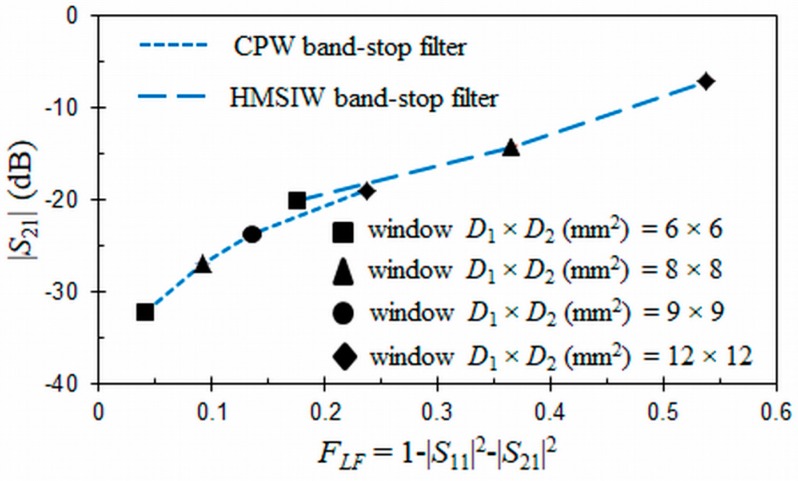
Rejection levels (in dB) at the resonant frequency $f_{0}$ for the proposed CPW and HMISW band-stop filters as a function of the forward loss factors and different open windows $D_{1}\times D_{2}$.

Finally, Figure 6 represents rejections levels (|*S*_21_| in dB) obtained at the resonance frequency $f_{0}$ of the proposed CPW and HMSIW band-stop filters as a function of the forward loss factors and different sizes of the open window $D_{1}\times D_{2}$. These results were obtained by means of EM simulations, neglecting metallic and dielectric losses. The dimensions of the CPW and HMSIW structures loaded with an ORR and the permittivity of the substrate are the same as those included in the captions of Figure 4 and Figure 5. In Figure 6, it can be noted that the rejection level at $f_{0}$ of the CPW band-stop filter decreases from −18.9 dB to −32.2 dB as the size of the open window $D_{1}\times D_{2}$ is reduced from 12×12 mm^2^ to 6×6 mm^2^, confirming the previous premise. The radiation losses decrease as the open window $D_{1}\times D_{2}$ is reduced. This same trend is observed for the HMSIW band-stop filter, with a rejection level variation from −7.1 dB to −20 dB. As a consequence of this analysis, a compromise was adopted for the size of the open window with the purpose to minimize the radiation losses at the main and spurious resonances, choosing an intermediate value for the open window of the CPW and HMSIW structures.

**Figure 7:**
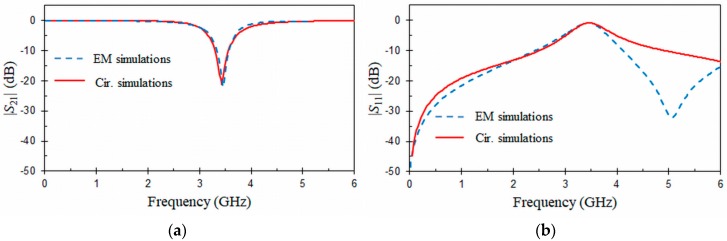
EM and circuit frequency responses for the CPW structure loaded with an ORR cell (**a**) |*S*_21_| (dB) and (**b**) |*S*_11_| (dB). Dimensions (mm) and permittivity: *r* =1.9, *c* = 0.3, *g* = 0.2, *D*_1_ × *D*_2_ = 9 × 9, *W* = 0.374, *S* = 0.163, *L*_1_ = 5.5, *t* = 0.017, *h* = 0.635, and *ε_r_* = 10.2 (tg*δ* = 0).

**Figure 8:**
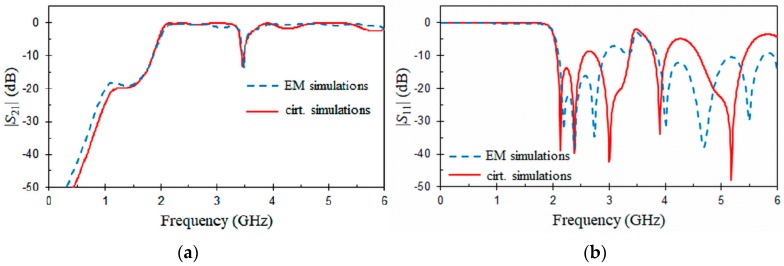
EM and circuit frequency responses for the HMSIW structure loaded with an ORR cell (**a**) |*S*_21_| (dB) and (**b**) |*S*_11_| (dB). Dimensions (mm) and permittivity: *r* = 1.9, *c* = 0.3, *g* = 0.2, *D*_1_ × *D*_2_ = 8 × 8, *W_HMSIW_* = 11.65, *s* = 1.2, *d* = 0.6, *W_m_* = 0.594, *W_t_* = 4.66, *L*_1_ = 5, *L*_2_ = 11, *L_t_* = 28.95, *t* = 0.017, *h* = 0.635, and ε*_r_* = 10.2 (tg*δ* = 0).

Figure 7 and Figure 8 show, respectively, EM simulation and equivalent circuit results for the CPW and HMSIW structures loaded with an ORR. These frequency responses were obtained by means of commercial simulators (Ansys HFSS, Keysight ADS). The dimensions of the CPW and HMSIW structures loaded with an ORR and the relative permittivity of the substrate used in the simulations are included in the captions of Figure 7 and Figure 8. Metallic losses were taken into account in the simulations, considering a copper thickness of *t* = 0.017 mm for the conductors. The elements $L_{0}$ = 5.3 nH and $C_{0}$ = 0.4 pF of the equivalent circuits (Figure 1b and Figure 2c) were obtained by using Equations (1)–(3) and the dimensions of the ORR. The half-mode substrate integrated waveguides (HMSIWs) filled with a substrate $\varepsilon_{r}$ in Figure 2c were simulated with the circuit simulator (Keysight ADS) by using the RWG (rectangular waveguide) model [46]. As it can be seen in Figure 7, EM simulations (dashed line) of the CPW structure loaded with an ORR exhibit typical stop-band behavior. At the resonant frequency $f_{0}$ = 3.46 GHz of the ORR, a transmission zero occurs. Return loss, insertion loss, and 3 dB stop-band bandwidth at $f_{0}$ = 3.46 GHz are, respectively, *RL* = 0.9 dB, *IL* = 21 dB, and $\Delta f_{0}$ = 0.7 GHz (20.2%). The previous data and equation (3) provide a quality factor $Q_{u}$ = 50. As expected, insertion loss (*IL* = 21 dB) is slightly lower than the rejection level obtained in Figure 6 (23.7 dB for an open window $D_{1}\times D_{2}$ = 9 × 9 mm^2^), due to the conductor loss considered in the EM simulations. In the passband at $f_{0}/3$, insertion loss is 0.1 dB, which is only 2 times higher than for a 50 Ω CPW line with the same length. A good agreement between circuit (solid line) and EM (dashed line) simulations for $\left|S_{21}\right|$ (Figure 7a) is obtained. However, discrepancies appear for $\left|S_{11}\right|$ (Figure 7b) at frequencies above 4 GHz. This is due to the simplification of the equivalent circuit model in which high-order effects are not considered, and because the parameter $S_{11}$ is more sensitive to these effects than the parameter $S_{21}$. Similar comments between EM and circuit simulations can be made for the HMSIW structure loaded with an ORR (Figure 8). In Figure 8, two behaviors can be observed for the EM simulations (dashed line). The first characteristic is relative to the high-pass frequency response due to the HMSIW line, which has a cut-off frequency at $f_{c}$ = 2 GHz. The second behavior is located in the pass-band of the HMSIW, where a transmission zero can be observed as a consequence of the ORR resonance. At $f_{0}$ = 3.49 GHz, an electric short to ground is produced and the injected signal is reflected back to the input port. Return loss, insertion loss, and 3 dB stop-band bandwidth at $f_{0}$ = 3.49 GHz are, respectively, *RL* = 2.2 dB, *IL* = 14 dB, and $\Delta f_{0}$ = 0.19 GHz (5.4%). These data and Equation (3) give an unloaded quality factor $Q_{u}$ = 82. In a similar way as for the CPW structure, insertion loss (*IL* = 14 dB) for the HMSIW structure is slightly lower than the rejection level obtained in Figure 6 (14.7 dB for an open window $D_{1}\times D_{2}$ = 8 × 8 mm^2^), due to the conductor loss considered in the EM simulations. In Figure 8, frequency response (solid line) obtained with the equivalent circuit also exhibits a high-pass behavior with a transmission zero in its pass-band.

### 2.3. Tunable CPW and HMSIW Band-Stop Filters Using VLORR

**Figure 9:**
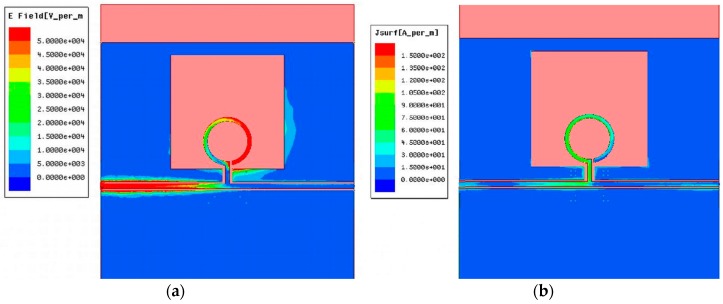
Distribution of the electric field (**a**) and current (**b**) for the CPW structure loaded with an ORR at the resonant frequency *f*_0_ = 3.46 GHz.

**Figure 10:**
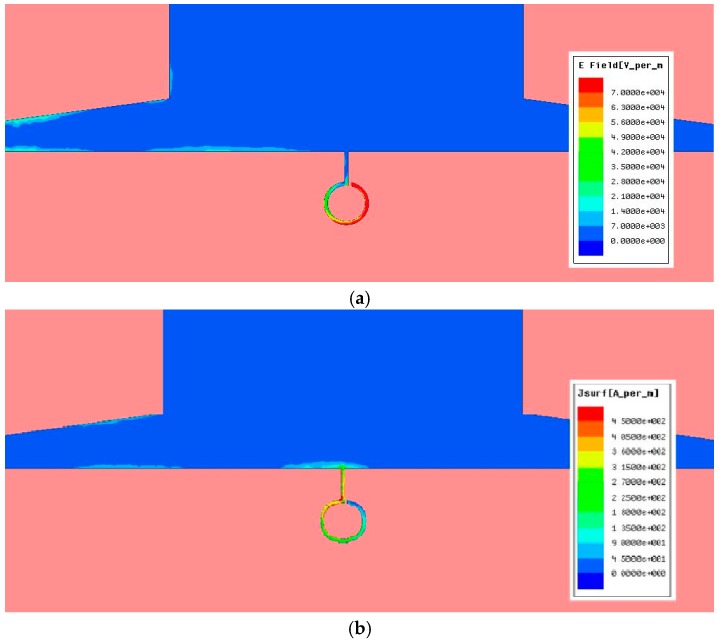
Distribution of the electric field (**a**) and current (**b**) for the HMSIW structure loaded with an ORR at the resonant frequency *f*_0_ = 3.49 GHz.

The resonant frequency $f_{0}$ of the CPW and HMSIW band-stop filters can be electronically controlled by inserting a varactor diode as tuning element. EM simulations have shown that the optimum location to have the widest tuning range is to connect the varactor diode between the middle point of the open ring and ground as in Figure 1a and Figure 2a. This is because at the resonance $f_{0}$ the current and electric field distributions (Figure 9 and Figure 10) are concentrated on the first and second half of the open ring, respectively. It can also be observed that at the resonance $f_{0}$, the electric field and current distributions are very similar in both structures. The capacitance of the varactor diode is controlled by applying a reverse DC bias voltage. Thus, an *LC* DC bias network was added to the varactor-loaded open ring resonator (VLORR). In Figure 1a and Figure 2a, the cathode of the varactor is located at the middle point of the open ring, while the anode is connected to ground through the decoupling capacitance *C* = 1 nF. To prevent the RF signal of the VLORR from perturbing the DC bias network, a choke inductance is inserted at the varactor anode. The value of this inductance was chosen as *L* = 330 nH. The simplified RF equivalent circuits of this varactor-loaded open ring resonator (VLORR) in CPW and HMSIW structures become the ones shown in Figure 1b and Figure 2c, considering the variable capacitance $C_{V}$ as the ideal model of the varactor diode, the field concentration in the open ring, and the above arrangement. Omitting $R_{0}$, the total capacitance of the CPW and HMSIW structure loaded with a VLORR is $C_{T}=C_{0}+C_{V}$, which represents the fixed capacitance $C_{0}$ of the ORR and the variable capacitance $C_{V}$ of the varactor diode. The resonant frequency $f_{0}$ of the CPW and HMSIW band-stop filters is varied in a controlled way through the variable capacitance $C_{V}$ of the varactor diode. Therefore, this diode works as a tuning component, with its reverse bias acting as control signal. In this work we have used a hyperabrupt varactor diode made of GaAs by Aeroflex/Metelix (MGV125-09). The capacitance $C_{V}$ of this diode varies from 3.4 pF to 0.07 pF when the reverse bias changes from 0 V to 22 V.

## 3. Fabrication and Results

The proposed VLORR cell presents an interesting application in the design of tunable band-stop filters. Thus, first- and second-order tunable CPW band-stop filters, and a tunable first-order HMSIW band-stop filter, were fabricated by means of prototyping laser (LPKF protolaser S) and milling (LPKF protomat S62) machines on a Rogers RO3010 substrate with the following properties and dimensions: $\varepsilon_{r}=10.2$, tg*δ* = 0.0023 at 10 GHz, substrate thickness *h* = 0.635 mm, and copper thickness *t* = 0.017 mm.

**Figure 11:**
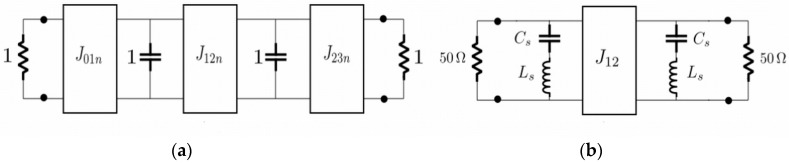
Equivalent circuits used for the synthesis of the second order band-stop filter. (**a**) Normalized low-pass prototype with admittance inverters; (**b**) final circuit after performing the low-pass to band-stop frequency transformation. Normalized loads are absorbed with first and last inverters and the whole circuit is scaled for 50 Ω ports impedance.

The second order band-stop filter was design for a center frequency of $f_{0}$ = 2.53 GHz and nominal bandwidth of BW = 1100 MHz, with a return loss level in the passbands of *RL* = 1.2 dB. For the synthesis of the filter, the regular procedure was applied starting from the low-pass prototype. Admittance inverters are used to convert the two lumped elements of this low-pass prototype into capacitors connected to ground, as show in Figure 11a. The relation between the admittance inverters and the prototype elements is:

(5) $$J_{i,i+1,n}=\frac{1}{\sqrt{g_{i}\;g_{i+1}}},\;i=0,1,2$$

where $g_{i}$ are the low-pass prototype elements. A standard low-pass to band-stop transformation is then applied [10], to convert the capacitors into two series resonators connected to ground. The normalized load impedances are absorbed with the first and last admittance inverters, and the whole circuit is scaled to have reference port impedances of $Z_{ref}=50\;\Omega$, obtaining the circuit shown in Figure 11b. After all these operations, the resulting components of the circuit are obtained with:

(6) $$C_{s}=\frac{FBW}{2\pi f_{0}J_{01n}^{2}Z_{ref}}$$

(7) $$L_{s}=\frac{1}{{\left(2\pi f_{0}\right)}^{2}C_{s}}$$

(8) $$J_{12}=\frac{J_{12n}}{J_{01n}^{2}Z_{ref}}$$

where FBW is the fractional bandwidth of the filter. The last transformation involves the implementation of the remaining ideal inverter ($J_{12}$) with a quarter wavelength transmission line transformer. After this process, the value of the elements of the resonators are $L_{s}$ = 6.2 nH and $C_{s}$ = 0.64 pF, while the characteristic impedance of the quarter wavelength transformer is $Z_{c}=38.7\;\Omega$.

**Figure 12:**
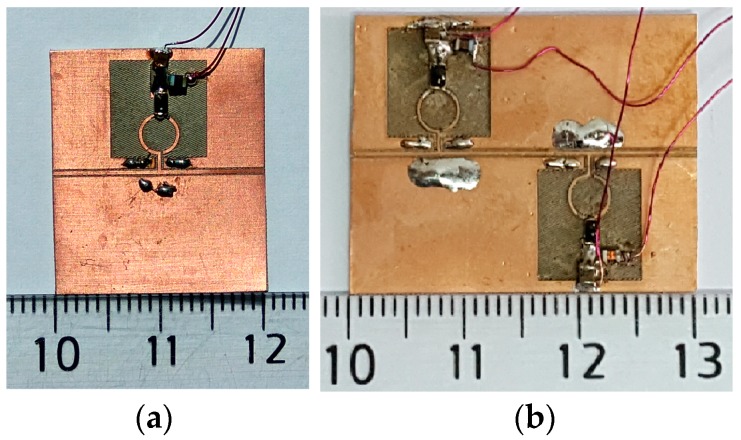
Photographs of the fabricated (**a**) first-order tunable CPW band-stop filter and (**b**) second-order tunable CPW band-stop filter.

**Figure 13:**
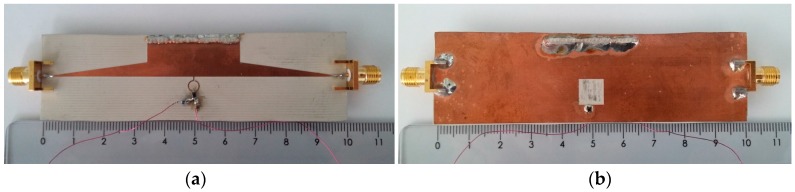
Photographs of the fabricated tunable HMSIW band-stop filter (**a**) top view and (**b**) bottom view.

The photographs of the first- and second-order tunable CPW band-stop filters are shown in Figure 12, while the photograph of the tunable HMSIW band-stop filter is depicted in Figure 13. A 50 Ω Anritsu universal 3680 K test fixture and coaxial-to-microstrip transitions (SMA) were used to carry out the measurements of the CPW and HMSIW filters, respectively. Measurements were performed by means of a vector network analyzer (R&S ZVA) between 0.01 GHz and 4 GHz.

**Figure 14:**
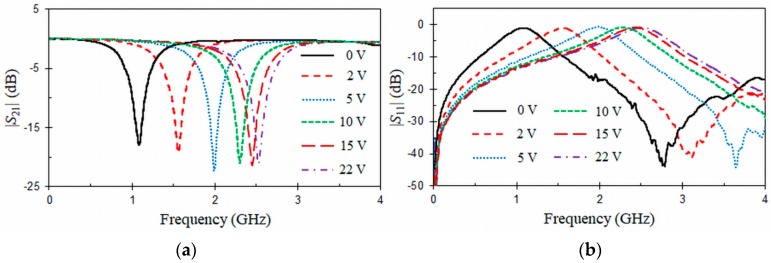
Measured (**a**) |*S*_21_| (dB) and (**b**) |*S*_11_| (dB) of the first-order tunable CPW band-stop filter under different DC bias.

**Figure 15:**
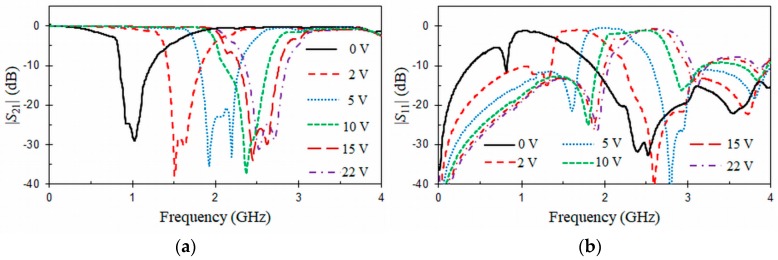
Measured (**a**) |*S*_21_| (dB) and (**b**) |*S*_11_| (dB) of the second-order tunable CPW band-stop filter under different DC bias.

Figure 14 and Figure 15 show, respectively, the measured *S*-parameters obtained for the first- and the second-order tunable CPW band-stop filters biased with different reverse DC voltages. In Figure 14, it can be seen that the resulting stop-band for the CPW structure loaded with a VLORR is tuned. The transmission zero (Figure 14a) at the resonant frequency $f_{0}$ of the VLORR cell varies from 2.53 GHz to 1 GHz as the capacitance of the varactor diode increases for a reverse $V_{bias}$ voltage from 22 V to 0 V. In this tuning range (1 GHz–2.53 GHz), the insertion and return losses at $f_{0}$ are, respectively, above *IL* = 18 dB and below *RL* = 1.1 dB. The varactor diode has little effect on the insertion loss. In the passband at $f_{0}/3$, insertion loss is lower than 0.16 dB for all the $V_{bias}$ range, which is around 3 times higher than a simulated 50 Ω CPW line with the same length. In comparison, this value was 2 times higher for a CPW band-stop filter without varactor (Figure 7). At the resonant frequency $f_{0}$, insertion loss is quasi-constant in the tuning range and close to that obtained by EM simulations for the CPW band-stop filter loaded with an ORR (Figure 7). A constant absolute bandwidth at 10 dB of $\Delta f_{10\mathrm{dB}}$ = 0.18 GHz can also be highlighted. This is due to the inductance value $L_{0}$ of the VLORR, which remains approximately constant along all the tuning range. Table 1 compares different tunable CPW band-stop filters loaded with varactor diodes. In this table, $\lambda_{0}$ is free-space wavelength at the center frequency of the tuning range. The proposed first-order tunable CPW band-stop filter has a better absolute tuning range ($\Delta f_{0}$ = 1.53 GHz) than any other design. In terms of size, it is more compact than the other structures, except for the designs presented in [26,27], although these ones use two meander lines and two rings (open split ring resonator, OSRR) while our design needs only one ring. Additionally, the present design and the one in [27] employ the lowest number of diodes and have a constant absolute bandwidth. From the compared frequency responses, the one in [26] (Figure 8) also presents a constant absolute bandwidth. Figure 15 depicts the measured *S*-parameters for the second-order tunable CPW band-stop filter biased with different reverse DC voltages. As expected, improved rejection at the resonant frequency $f_{0}$ is obtained with a higher-order tunable CPW band-stop filter. The tuning range is the same as the previous first-order tunable CPW band-stop filter. However, the constant absolute bandwidth behavior is lost, because the CPW section of length $L_{S}$ = 13.126 mm (λ/4 at *f* = 2.53 GHz), acting as impedance inverter between the centers of the two cascaded VLORRs, is not tuned.

**Figure 16:**
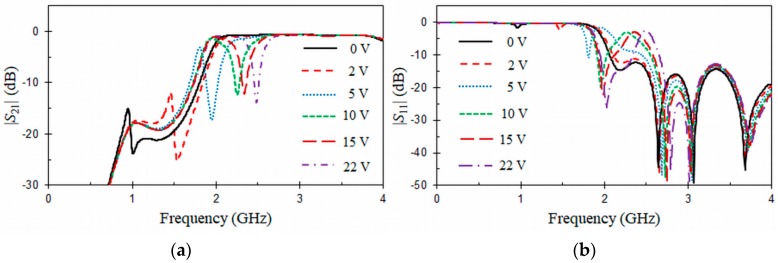
Measured (**a**) |*S*_21_| (dB) and (**b**) |*S*_11_| (dB) +of the tunable HMSIW band-stop filter under different DC bias.

Measured *S*-parameters frequency responses of the tunable HMSIW band-stop filter biased with different reverse DC voltages are represented in Figure 16. As it can be seen, two behaviors can be observed. The first one depends on the HMSIW line, which has a typical high-pass frequency response with a cut-off frequency around $f_{c}$ = 2 GHz. The second behavior is due to the resonance of the VLORR cell, which is moved from 2.5 GHz to 1 GHz as the capacitance of the varactor diode increases for a reverse $V_{bias}$ voltage from 22 V to 0 V. The resonance of the VLORR cell implements a transmission zero (Figure 16a) above the cut-off frequency of the HMSIW line (*f* > $f_{c}$) for bias voltages above 5 V. However, the transmission zero falls below the cut-off frequency $f_{c}$ for bias voltages below 5 V, and therefore it is not anymore useful in that range. In the pass-band of the HMSIW structure (*f* > 2 GHz), the insertion and return losses at $f_{0}$ are, respectively, above *IL* = 12.3 dB and below *RL* = 3.1 dB. It can also be observed in Figure 16a that the absolute bandwidth at 10 dB is constant $\Delta f_{10\mathrm{dB}}$ = 0.05 GHz, because the inductance value of the VLORR remains approximately constant along the tuning range above the cut-off frequency (*f* > 2 GHz). Table 2 compares different tunable SIW/HMSIW band-stop filters loaded with varactor diodes. In this table, $\lambda_{0}$ is free-space wavelength at the center frequency of the tuning range. The tunable band-stop filters designed in [30,37,38] are, respectively, based on 5 cascaded cells and two coaxial-cavity resonators. The first-order tunable HMSIW band-stop filter proposed here presents a better absolute tuning range ($\Delta f_{0}$ = 1.5 GHz) and a more compact size than any other design presented in Table 2. Additionally, the present design uses only one varactor diode. From the compared designs, the one in [29] (Figure 3) can demonstrate also the use of only one varactor diode.

In contrast to the OSRR (VLOSRR) presented in [12,13,27], whose behavior as pass-band or stop-band filter depends on whether it is connected to a microstrip or CPW section, our ORR (VLORR) is more versatile, because it achieves impressive tunable stop-band characteristics regardless of the type of transmission line to which it is connected (either CPW or HMSIW).

## 4. Conclusions

First- and second-order tunable CPW band-stop filters and a first-order tunable HMSIW band-stop filter using varactor-loaded open ring resonators (VLORRs) have been implemented. The equivalent circuits of the CPW and HMSIW loaded with a VLORR have been derived and the radiation effects have been analyzed. The first-order tunable CPW and HMSIW filters can be designed with a forward loss factor lower than 5% and 18%, respectively, at the main resonance. The use of the reverse-biased varactor diode allows the control of the stop-band frequency of the CPW and HMSIW filters using VLORR cells. The measured tunable CPW and HMSIW band-stop filters present a compact size and high performance and, therefore, they can be useful in emerging multiband and multifunction communication systems.

## Figures and Tables

**Table 1:** Comparison of different tunable CPW band-stop filters.

References	Tuning Range in GHz (%)	No. of Diodes	Absolute BW	2-D Size
[9] Figure 7	1.69–2.16 (24.4%)	2	Not constant	0.064 *λ*_0_ × 0.16 *λ*_0_
[23] Figure 8b	3.35–4.05 (18.9%)	2	Not constant	0.148 *λ*_0_ × 0.074 *λ*_0_
[24] Figure 5	2.31–2.19 (5.3%)	4	Not constant	0.29 *λ*_0_ × 0.375 *λ*_0_
[25] Figure 10	3.15–3.9 (21.3%)	3	Not constant	0.094 *λ*_0_ × 0.071 *λ*_0_
[26] Figure 8	0.51–1.76 (110.1%)	2	Constant	0.015 *λ*_0_ × 0.034 *λ*_0_
[27] Figure 3	0.6–1.6 (90.9%)	1	Constant	0.021 *λ*_0_ × 0.022 *λ*_0_
This work Figure 12	1–2.53 (86.6%)	1	Constant	0.054 *λ*_0_ × 0.054 *λ*_0_

**Table 2:** Comparison of different tunable SIW/HMSIW band-stop filters.

Refs.	Tuning Range in GHz (%)	No. of Diodes	Absolute BW	2-D Size
[28] Figure 7a	10.05–10.26 (2.1%)	2	Constant	0.5 $\sqrt{\varepsilon_{ref}}$ *λ*_0_ × 0.5 $\sqrt{\varepsilon_{ref}}$
[29] Figure 3	5.32–5.54 (4.1%)	1	Constant	0.083 *λ*_0_ × 0.163 *λ*_0_
[29] Figure 5	3.5–4.1 (15.8%)	2	Constant	0.5 $\sqrt{\varepsilon_{ref}}$ *λ*_0_ × not defined
[30] Figure 21	2.39–4.39 (69.2%)	5	Not constant	0.483 *λ*_0_ × 0.011 *λ*_0_
[37] Figure 7	0.56–1.18 (41.6%)	64	Not constant	0.228 *λ*_0_ × 0.059 *λ*_0_
[38] Figure 5	0.77–1.25 (47.5%)	8	Not constant	0.154 *λ*_0_ × 0.101 *λ*_0_
This work Figure 13	1–2.5 (85.7%)	1	Constant	0.047 *λ*_0_ × 0.047 *λ*_0_
